# Alternative splicing of MR1 regulates antigen presentation to MAIT cells

**DOI:** 10.1038/s41598-020-72394-9

**Published:** 2020-09-22

**Authors:** Gitanjali A. Narayanan, Abhinav Nellore, Jessica Tran, Aneta H. Worley, Erin W. Meermeier, Elham Karamooz, Megan E. Huber, Regina Kurapova, Fikadu G. Tafesse, Melanie J. Harriff, David M. Lewinsohn

**Affiliations:** 1 Department of Biomedical Engineering, Oregon Health and Science University, Portland, OR USA; 2 Department of Computational Biology, Oregon Health and Science University, Portland, OR USA; 3 VA Portland Health System, Portland, OR USA; 4 Department of Pulmonary and Critical Care Medicine, Oregon Health and Science University, Portland, OR USA; 5 Department of Molecular Microbiology and Immunology, Oregon Health and Science University, Portland, OR USA

**Keywords:** Immunology, Molecular biology

## Abstract

Mucosal Associated Invariant T (MAIT) cells can sense intracellular infection by a broad array of pathogens. These cells are activated upon encountering microbial antigen(s) displayed by MR1 on the surface of an infected cell. Human MR1 undergoes alternative splicing. The full-length isoform, MR1A, can activate MAIT cells, while the function of the isoforms, MR1B and MR1C, are incompletely understood. In this report, we sought to characterize the expression and function of these splice variants. Using a transcriptomic analysis in conjunction with qPCR, we find that that MR1A and MR1B transcripts are widely expressed. However only MR1A can present mycobacterial antigen to MAIT cells. Coexpression of MR1B with MR1A decreases MAIT cell activation following bacterial infection. Additionally, expression of MR1B prior to MR1A lowers total MR1A abundance, suggesting competition between MR1A and MR1B for either ligands or chaperones required for folding and/or trafficking. Finally, we evaluated CD4/CD8 double positive thymocytes expressing surface MR1. Here, we find that relative expression of *MR1A/MR1B* transcript is associated with the prevalence of MR1 + CD4/CD8 cells in the thymus. Our results suggest alternative splicing of MR1 represents a means of regulating MAIT activation in response to microbial ligand(s).

## Introduction

MR1-restricted T (MAIT) cells are a subset of CD8 αβ T cells that represent 1–10% of circulating T cells. They are enriched in mucosal sites, including the skin, intestinal mucosa, and lung, and they can serve to sense intracellular microbial infection^1–3^. MAIT cells produce the effector cytokines IFN-γ and TNF-α following encounter with infected cells^2,3^. MAIT cells respond to a wide array of microbes including, but not limited to, *Mycobacterium tuberculosis* (Mtb), *Escherichia coli (E. coli)*, *Salmonella enterica* serovar Typhimurium, *Candida albicans (C. albicans)*, *Legionella pneumophila* , and *Streptococcus* species^2–6^. While the prevalence and phenotype of MAIT cells in mice is distinct from that in humans, mice lacking MAIT cells had reduced capacity to control infection with *F. tularensis*, *Mycobacteria bovis* BCG (BCG), *Klebsiella pneumoniae, and Legionella longbeachae*^6–9^. In some cases, MAIT cells have been associated with the maturation of dendritic cells (DC), which in turn play a role in the recruitment of activated Major Histocompatibility Complex (MHC) Class I and Class II restricted T cells^6,9^. Therefore, MAIT cells represent an important mechanism by which the immune system senses and responds to microbial infection.

MAIT cells recognize microbial antigen processed and presented by the MHC Class I related molecule, MR1^10^. MR1, though similar in many ways to canonical Class I molecules, has distinct features that render it uniquely suited to present microbial ligands. *MR1* transcript is expressed in all nucleated cells; however unlike MHC Class I molecules, which are constitutively detected on the cell surface, MR1 resides in the endoplasmic reticulum (ER) as well as late endosomal vesicles^11,12^. Following infection, MR1 binds microbial ligand, and this complex is thought to traffic to the cell surface to stimulate MAIT cells^11,12^. We have previously shown that MR1 mediated antigen presentation is dependent on the vesicular trafficking proteins Syntaxin18 and VAMP4^13^. More recently, we have observed that distinct trafficking pathways exist to present endogenous and exogenous mycobacterial antigen by MR1 to stimulate MAIT cells^13,14^.

While MHC Class I molecules traditionally present peptides to stimulate CD8 + T cell responses, MR1 binds and presents microbial small molecule metabolites to MAIT cells^10,15^. These antigens were first described as intermediates in the riboflavin synthesis pathway, but recent reports have highlighted the increasing diversity of the MAIT ligand repertoire^15–17^. For example, MR1 also presents antigen(s) from *S. pyogenes*, a bacteria that cannot synthesize riboflavin^4^. In silico screens showed that MR1 can bind a range of synthetic compounds, including commonly prescribed pharmaceuticals^16^, We have recently shown through metabolomics analysis that MAIT stimulatory antigens include ligands distinct from those generated in the riboflavin synthesis pathway^5^.

The underlying gene organization of MR1 is distinct from canonical MHC molecules. *MR1* is non polymorphic, highly conserved across species and individuals, with the transcript ubiquitously expressed^18–20^. *MR1* pre-mRNA undergoes alternative splicing to produce multiple isoforms, which have been demonstrated at the transcript level to be expressed in human tissues and cell lines^21^. The structure of MR1 is similar to that of MHC Class I molecules, with α1 and α2 domains that bind ligand, an α3 domain that interacts with β2-microglobulin, and a transmembrane domain for surface expression^21,22^. The full length isoform, MR1A, contains all encoded exons and can stimulate MAIT cells. The shorter isoform MR1B lacks the α3 domain but does encode the ligand binding and transmembrane domains. The function of MR1B is remains incompletely understood. Overexpression of MR1B in a fibrosarcoma model suggested a functional role for MR1B in stimulating MAIT cells following infection with *E. coli*^23^. MR1C is a putative soluble isoform, lacking both the α3 and transmembrane domains, however, there are no reports on its expression or function.

Here, we sought to determine the role of MR1 isoforms in the presentation of microbial ligand to MAIT cells. We show *MR1A and MR1B* transcripts are detectable across human tissues, with considerable variation in isoform expression among donors and tissues. We developed a lung epithelial cell line deficient in *MR1* and utilized this system to show that MR1B can antagonize MR1A in the presentation and/or processing of mycobacterial antigen(s). While MR1A is observed in the ER and vesicular compartments, MR1B appears to reside primarily in intracellular vesicles. Finally, we show that surface expression of MR1A on CD4 + CD8 + MR1 expressing thymocytes is associated with relative abundance of MAIT cells in the thymus**.** Taken together, our results suggest that the splice variant MR1B can regulate the response of MAIT cells to intracellular infection.

## Results

### MR1A and MR1B are ubiquitously expressed

**Figure 1:**
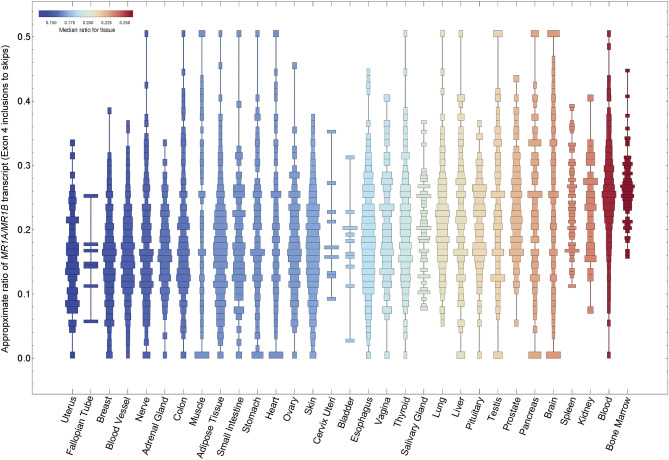
Distribution of relative *MR1A/MR1B* transcript across the GTEx dataset. Snaptron was used to query human transcriptome data from the publicly available GTEx dataset of non-malignant human tissues. Relative *MR1A/MR1B* mRNA expression was measured by quantifying junctional inclusion ratios of exon 3 inclusions (*MR1A*) to exon 3 skips (*MR1B*). We depict histograms of the ratio of *MR1A/MR1B* transcript expression in order of increasing mean expression.

To study expression of the MR1 splice variants, we used Snaptron, a tool for exploring exon-exon junction expression across thousands of publicly available RNA sequencing (RNA-seq) samples^24,25^. We analyzed exon-exon junctions corresponding to inclusion or exclusion of exon 4 to distinguish *MR1A* from *MR1B*, using nearly 10,000 RNA-seq samples from 31 non-diseased tissue sites in over 500 deceased individuals from the Genotype Tissue Expression (GTEx) project (see Materials and Methods)^26^. Here, we show that both *MR1A* and *MR1B* are expressed across human tissues, as seen in prior, non-quantitative studies (Fig. 1)^18,21,27^. The ratio of *MR1A* to *MR1B* varied across tissues, with higher relative *MR1A* transcript observed in blood, bone marrow, liver, and lung, and lower relative *MR1A* observed in uterine cervix, breast, small intestine, and colon*.* Interestingly, we observed that in all the tissues queried, the *MR1A/MR1B* ratio was consistently less than 0.5, suggesting that all the tissues express higher relative *MR1B* than *MR1A.* As these data are from the total mRNA for a given tissue, this analysis does not take into account diversity of the individual cell types that comprise each tissue.

### Quantitative RT-PCR analysis reveals differing levels of MR1 splice variants in antigen presenting cells

**Figure 2:**
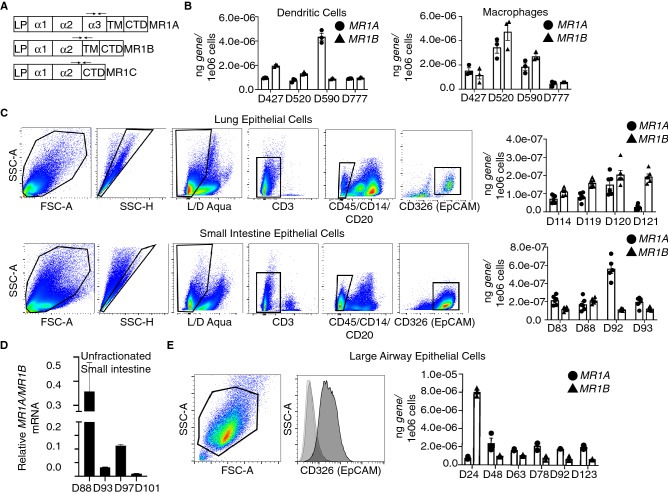
*MR1A* and *MR1B* mRNA are detectable in antigen presenting cells isolated from human tissues. Quantitative real-time PCR (qRT-PCR) primers were designed to specifically amplify either *MR1A* or *MR1B* transcript*.* (**A**). In brief: RNA was isolated from 5e04 cells and qRT-PCR was performed to quantify the absolute amounts of *MR1A* or *MR1B* mRNA as measured by a standard curve designed to be specific for each isoform. (**B**) qRT-PCR performed on monocyte-derived macrophages and monocyte-derived dendritic cells generated from human PBMC (n = triplicate wells from one experiment, error bars represent mean and standard deviation from the mean). (**C**) Human lung parenchyma (top) and small intestine lamina propria (bottom) were stained with antibodies against CD45, CD20, CD14 and CD326 (EpCAM). CD45- CD20- CD14- CD326 + cells were sorted for mRNA isolation and cDNA synthesis. Briefly, cells were gated sequentially on cell shape, singlet cells, live, CD3-, CD45-/CD14-/CD20-, CD326 + (Epithelial Cells). qRT-PCR was performed as described twice in triplicate (n = 6, error bars represent mean and standard deviation from the mean). (**D**) qRT-PCR was performed on unfractionated small intestine from 4 donors. Experiment was performed twice with triplicate wells. (n = 6, error bars represent mean and standard error of the mean of 6 replicates) (**E**) Human Large Airway Epithelial Cells (LAEC) were isolated and cultured from the upper airway and stained with an antibody to either isotype (IgG2b) or CD326 (EpCAM) to verify surface expression of EpCAM. mRNA was isolated and qRT-PCR performed as described. (n = 3 replicates from 1 experiment, error bars represent mean and standard deviation from the mean).

To explore the relative MR1 splice variant expression specifically in primary cells, we focused on macrophages, monocyte derived DC as well as epithelial cells, all of which can present Mtb-derived antigens to MAIT cells^12^. Using quantitative real-time PCR (qRT-PCR), we generated a standard curve for each splice variant by using plasmids designed to be specific to each isoform based on unique exon-exon junctions (Fig. 2A). We found that these primers were specific in that we did not observe amplification in a Bronchial epithelial cell line (Beas2B) lacking MR1, and that transfection with plasmids encoding each isoform were specifically amplified, with no amplification detected for the other splice variants. (Fig. 2A and Supplemental Fig. 1). In this manner, we were able to quantify absolute as well as relative amounts of the MR1 splice variants. Macrophages and DC were prepared from PBMC^28^. We observed that in both dendritic cells (DCs) and macrophages, both *MR1A* and *MR1B* transcripts were readily detected (Fig. 2B). To determine the expression of MR1 splice variants in primary human epithelial cells, we generated single cell suspensions from lung parenchyma and small intestine and subsequently sorted epithelial cells by EpCam (CD326) expression (Fig. 2C, top-Lung Epithelial Cells, bottom-Small Intestine Epithelial Cells). Using qRT-PCR, we found that the relative expression of *MR1A* was again similar to *MR1B*. Interestingly, both lung and small intestine epithelial cells expressed nearly 2-log lower levels of *MR1* splice variants than PBMC derived myeloid cells. We observed a discrepancy between the quantities of the MR1 isoforms in isolated epithelial cells from tissue versus bulk tissue from the RNA-seq analysis in Fig. 1. Therefore, we performed the same analysis on single cell suspensions derived from unfractionated small intestine tissue and observed ratios of MR1A/MR1B similar to what was observed in Fig. 1. (Fig. 2D). This indicates that the relative expression of *MR1A* and *MR1B* vary greatly between tissue types, as well as cell types within each tissue.

As primary large airway epithelial cells (LAEC) are capable of presenting mycobacterial antigen to stimulate MAIT cells, we prepared LAEC from human airway tissue, and confirmed that these cells expressed for EpCam (Fig. 2E)^2^. LAEC isolated from 5/6 donors expressed relatively more *MR1A* mRNA compared to *MR1B.* Overall transcript expression of these LAEC *MR1* splice variants was similar to levels observed in APCs generated from PBMC. *MR1A* mRNA levels were more consistent across donors, ranging from 1.0E−06 to 2.1E−06 ng, while *MR1B* transcript ranged from 2E−07 to 4E−06 ng. Of note, LAEC from one donor, D24, expressed considerably higher (fourfold) expression of *MR1B* than *MR1A*, relative to the LAEC prepared from the remaining donors which expressed 2–5 fold higher *MR1A* relative to *MR1B.* Taken together, these results confirm that both *MR1A* and *MR1B* transcript are expressed, but highlights heterogeneity both within tissues and across donors with regard to the expression of *MR1* splice variants.

### MR1B inhibits T cell activation by MR1A

**Figure 3:**
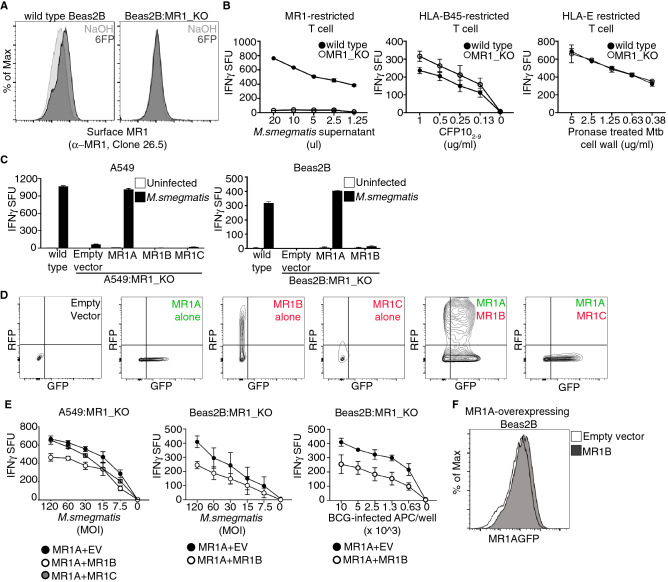
MR1B inhibits T cell activation by MR1A. Wild type Beas2B cells were transduced with a lentivirus targeting *MR1* using CRISPR/Cas9 gene editing to generate a Beas2B MR1 knockout cell line (Beas2B:MR1_KO). (**A**) Surface expression of MR1A assessed using flow cytometry of antibody staining to MR1A (α-MR1, 26.5) following exposure of wt Beas2B and Beas2B:MR1_KO cells to 6FP overnight. (**B**) wt Beas2B and Beas2B:MR1_KO cells were treated with either *M.smegmatis* supernatant (left), pronase treated Mtb cell wall (middle), or CFP10_2-9_ and (right) utilized to stimulate either a MR1-restricted T cell clone (left), an HLA-B45 restricted T cell clone (middle), or a HLA-E restricted T cell clone (right). IFN-γ production is measured by ELISpot and reported as IFN-γ spot forming units/5,000 T cells (IFN-γ SFU). Error bars represent mean and standard deviation of the mean from duplicate wells. Data are representative of > 3 independent experiments. (**C**) A549:MR1_KO and Beas2B:MR1_KO cells were transfected with plasmids encoding either MR1AGFP, MR1BRFP or MR1CRFP, or a pCI empty vector. Cells were infected overnight with *M. smegmatis* at a multiplicity of infection (MOI) of 3 and utilized as antigen presenting cells to stimulate MAIT production of IFN-γ in an ELISpot as described in (**B**). (z) Beas2B:MR1_KO cells were transfected as described and MR1BRFP or MR1CRFP expression was measured by detection of total RFP by flow cytometry. (**E**) A549:MR1_KO or Beas2B:MR1_KO cells were cotransfected with plasmids encoding MR1AGFP along with either the pCI empty vector, MR1BRFP, or MR1CRFP. (Left, Middle) Cells were infected for 1 h with *M. smegmatis* and used as in (**B**). MOI is indicated on x-axis. Data are pooled from at least 3 independent experiments with duplicate wells and error bars represent mean and error from n = 6 replicates. (Right) Beas2B:MR1_KO cells were infected with BCG overnight at MOI of 15, and used to stimulate IFN-γ production by MAIT cells. Number of infected APCs used are indicated on x-axis. Data are pooled from at least 2 independent experiments with duplicate wells, and error bars represent mean and error of n = 4 replicates from 2 independent experiments. (**F**) Beas2B overexpressing MR1AGFP were transiently transfected with either the pCI empty vector or pCI_MR1BRFP. Flow cytometric analysis was performed to quantify total MR1A expression, as measured by detection of GFP, following transfection. Data are representative of > 2 independent experiments.

We next sought to assess the function of the MR1 isoforms in the absence of endogenous MR1. As previously described in A549 cells, CRISPR/Cas9 was used to generate a bronchial epithelial cell line (Beas2B) that lacked the gene for *MR1* (Beas2B:MR1_KO)^29^. To identify MR1 deficient cells, we performed limiting dilution cloning, and then screened for the absence of 6FP induced MR1 surface stabilization (Fig. 3A)^30^. Functionally, we found that these clones could not stimulate an MR1-restricted clone in response to *M. smegmatis* infected APC (Fig. 3B). In contrast, stimulation of both HLA-B45 and HLA-E restricted clones was not decreased, and in the case of HLA-B45, was slightly, but not significantly, increased, indicating that the knockout was specific to MR1 and not to MHC Class I in general (Fig. 3B).

We next generated expression vectors containing the genes for *MR1A*, *MR1B,* or *MR1C* under the control of the CMV promoter. To distinguish MR1A from MR1B or MR1C following transfection, we tagged MR1A with GFP and MR1B and MR1C with RFP (pCI:MR1AGFP, pCI:MR1BRFP, pCI:MR1CRFP). We then transfected these plasmids individually into Beas2B:MR1_KO and A549:MR1_KO cells and infected the cells with *M. smegmatis* overnight in order to stimulate MAIT cells (Fig. 3C). Flow cytometry was used to confirm protein expression both in single transfectants and co-transfected cells (Fig. 3D). While MR1C protein was detectable by flow cytometry, it was expressed at markedly lower levels than MR1B both in single and cotransfected samples (Fig. 3D). We verified that transcript of each isoform was expressed by performing qRT-PCR on cells transfected with pCI:MR1AGFP, pCI:MR1BRFP, pCI:MR1CRFP (Supplemental Fig. 1).

As described above, untransfected A549:MR1_KO and Beas2B:MR1_KO cells were incapable of stimulating MAIT cell production of IFN-γ^29^. Confirming the specific nature of the knockout, expression of pCI:MR1AGFP in both Beas2B:MR1_KO and A549:MR1_KO cells reconstituted MAIT cell dependent recognition of *M. smegmatis*. Transfection with the pCI empty vector, pCI:MR1BRFP or pCI:MR1CRFP did not result in MAIT production of IFN-γ (Fig. 3C).

As we found that neither MR1B nor MR1C could present antigen to MAIT cells, we postulated that these isoforms could work in concert with MR1A to modulate MAIT activation. To test this hypothesis, we coexpressed pCI:MR1AGFP with pCI:MR1BRFP or pCI:MR1CRFP in Beas2B:MR1_KO and A549:MR1_KO cells, and subsequently infected the cells with *M. smegmatis*. Coexpression of MR1A with MR1B resulted in a 40% decrease in MAIT cell activation in both cell lines with *M. smegmatis* infection (Fig. 3E, left, middle). However, coexpression of MR1C with MR1A did not lead to inhibition of MR1A-mediated presentation of *M.smegmatis-*derived ligand. While we observed inhibition of TCR activation when there was a 1:1 ratio of MR1B:MR1A, we observed a more marked inhibition of MR1A-dependent antigen presentation when we transfected a 2:1 ratio of MR1B:MR1A expressing plasmid, keeping the quantity of MR1A fixed (Supplemental Fig. 2). We selected this higher dose of MR1B for all subsequent experiments, as this quantity of plasmid was most similar to RNA data (Fig. 1). As antigens derived from *M. smegmatis* can be presented exogenously, we sought to test a mycobacteria for which recognition depends on intracellular infection^14^. As shown in Fig. 3E (right), MR1B associated inhibition was also observed following infection with BCG. Co-expression of MR1B or MR1C with MR1A resulted in a slight increase in constitutive MR1A surface protein abundance as measured by flow cytometry (Fig. 3F). Additionally, coexpression of MR1B with MR1A did not impede antigen presentation of peptide antigen by Beas2B:MR1_KO cells to HLA-B45 restricted T cells, indicating that the inhibition was specific to MR1 (Supplemental Fig. 3). Taken together, these results indicate that MR1B is functioning selectively to inhibit T cell activation by MR1A.

### MR1B resides in the same intracellular compartments as MR1A

**Figure 4:**
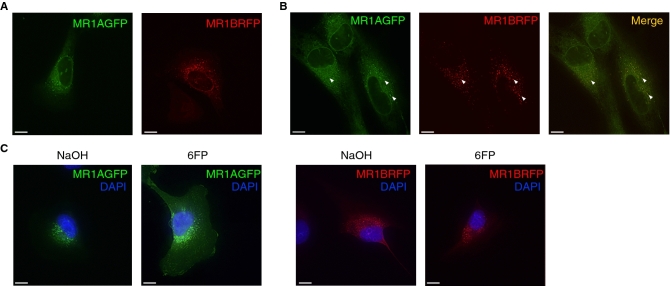
MR1A and MR1B reside in the same intracellular compartments. (**A**) Beas2B:MR1_KO cells were transfected with plasmids encoding either MR1AGFP or MR1BRFP. Live cell imaging was performed using a CoreDV microscope to detect intracellular MR1A (left) or MR1B (right). Scale bars represent 10 µm. (**B**) MR1A-overexpressing Beas2B cells were transfected with a plasmid encoding MR1BRFP and live cell imaging was performed as described in (**A**) to determine the subcellular localization of MR1A and MR1B. (left) MR1A alone, (middle) MR1B alone, right (merge). (**C**) Beas2B:MR1_KO cells were transfected with plasmids encoding either MR1AGFP or MR1BRFP, and subsequently treated with 50 µm 6FP or NaOH for 16 h. Cells were fixed for 20 min with 4% PFA and imaging was performed using a CoreDV microscope to determine localization of MR1A or MR1B with or without 6FP. DAPI nuclear stain was used to identify the nucleus. Scale bars represent 10 µm. Experiments were repeated 2–4 times with similar results.

In the absence of ligand, MR1A has been found to reside in the endoplasmic reticulum as well as intracellular vesicles^11,13,14^. The subcellular localization of MR1B is not fully understood, however, transient overexpression of MR1B in a fibrosarcoma cell line showed that MR1B is capable of forming stable homodimers at the cell surface^23^. However, these studies were performed in cell lines expressing endogenous MR1 isoforms. To elucidate the subcellular localization of MR1A and MR1B in knockout cell lines, we individually expressed pCI:MR1AGFP or pCI:MR1BRFP in Beas2B:MR1_KO cells and performed live cell imaging (Fig. 4A). MR1A (left) was detectable in both the endoplasmic reticulum as well as intracellular vesicles, as has been previously described^11,13,14^. As shown in Fig. 4A (right), MR1B had a similar intracellular distribution to MR1A, particularly localizing to intracellular vesicles. Co-expression of pCI:MR1AGFP and pCI:MR1BRFP in Beas2B:MR1_KO cells demonstrated association of MR1A and MR1B in similar compartments (Fig. 4B). This suggested that MR1B could depend on its close proximity to intracellular MR1A for the inhibition we observed. It has been previously established that translocation of MR1A to the cell surface is dependent on the availability of ligands such as 6FP^13,30^. As MR1A and MR1B reside in the same intracellular compartments, we postulated that MR1B could interfere with either the translocation of MR1A to the cell surface or the engagement with the MAIT T cell receptor on the cell surface. Beas2B:MR1_KO cells transfected with either pCI:MR1AGFP or pCI:MR1BRFP were exposed to 6FP overnight and imaging was performed to detect cell surface translocation. While we observed cell surface expression of MR1A, MR1B remained in intracellular vesicles (Fig. 4C). Therefore, these results suggest that MR1B-mediated antagonism is mediated intracellularly, prior to MR1A cell-surface translocation, rather than to directly block the MAIT TCR.

### MR1B expression limits abundance of MR1A protein

**Figure 5:**
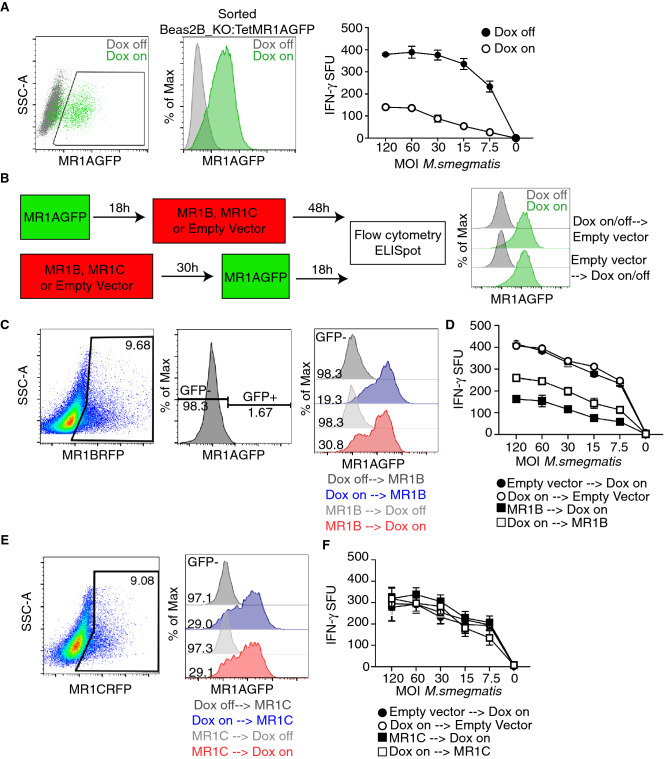
Prior MR1B expression decreases the abundance of MR1A protein. (**A**) Beas2B:MR1_KO cells were transduced with a lentivirus encoding a doxycycline-inducible MR1AGFP and sorted based on MR1AGFP expression following overnight administration of doxycycline. Beas2B_MR1KO_TetMR1AGFP cells were treated with doxycycline overnight and infected with *M.smegmatis* at a dose response of MOI for 1 h, which is indicated on x-axis. Infected cells were used as antigen presenting cells in an ELISpot assay as described earlier to stimulate MAIT production of IFN-γ. Data are representative of at least 3 independent experiments performed in duplicate with similar results. Error bars represent mean and standard deviation from the mean of one experiment. (**B**) Schematic of experiments to study the timing of MR1A expression with MR1B or MR1C. Briefly, doxycycline was added to Beas2B_MR1KO_TetMR1AGFP cells 18 h prior to or following the transfection with MR1BRFP, MR1CRFP or a pCI empty vector. The timing of transient transfection with MR1BRFP was fixed at 48 h for both conditions to ensure consistency with prior experiments. Doxycycline to induce expression of MR1A was administered for 18 h prior to transient transfection with pCI_MR1BRFP or pCI_MR1CRFP (top), OR ELISpot or flow cytometry to measure function or expression of MR1AGFP. MR1AGFP expression pre and post transfection with a pCI empty vector was measured by flow cytometry (right). (**C**) Transfected cells were assessed by flow cytometry, gated on MR1B expression using RFP (left), and then assessed for GFP expression, which was based on a dox-off control (second). Overlay histograms of MR1AGFP expression following MR1B (third) transfection as described in Fig. 6B with gMFI of MR1AGFP-negative condition reported. Doxycycline off transfection conditions are included as a control. Data are representative of at least 3 independent experiments with similar results. (**D**) ELISpot of cells transfected according to (B) and used as antigen presenting cells following infection with *M.smegmatis* at the indicated MOI (x-axis). Cells were incubated with a MAIT clone and IFN-γ production was measured. Data are pooled from 3 independent experiments performed in duplicate and error bars represent mean and standard deviation of replicates (n = 6). (**E**) Beas2B:MR1_KO cells were transfected with a plasmid expressing MR1CRFP. Transfected were assessed by flow cytometry, gated on MR1C expression using RFP (left), and then assessed for GFP expression, which was based on a dox-off control (second). Overlay histograms of MR1AGFP expression following MR1C (third) transfection as described in Fig. 6B with gMFI of MR1AGFP-negative condition reported. Doxycycline off transfection conditions are included as a control. Data are representative of at least 3 independent experiments with similar results. (**F**) ELISpot of cells transfected with pCI_MR1CRFP according to (B) and used as antigen presenting cells following infection with *M.smegmatis* at the indicated MOI (x-axis). Cells were incubated with a MAIT clone and IFN-γ production was measured. Data are pooled from 3 independent experiments performed in duplicate and error bars represent mean and standard deviation from the mean (n = 6).

In the experiments described above, both MR1A and MR1B were expressed under the control of the CMV promoter. However, as described above, both the abundance and relative expression of each isoform was highly variable. To control the timing and expression of MR1A, we developed a MR1A construct under the control of a doxycycline-dependent promotor. To accomplish this, we stably transduced Beas2B:MR1_KO cells with a lentivirus encoding a tetracycline-inducible MR1AGFP (Beas2B:MR1KO_Tet_MR1AGFP) and sorted the transduced cells based on GFP expression following overnight administration of doxycycline (Fig. 5A)^14^. We confirmed that these cells were capable of presenting *M. smegmatis*-derived antigen to MAIT cells upon overnight induction of MR1A expression with doxycycline. Using this cell line, we could control the expression of MR1A expression by administering doxycycline either before or after transfecting the cells with pCI:MR1BRFP (Fig. 5B-left). In all conditions we performed infections 48 h following MR1B transient transfection to ensure optimal expression of MR1B and consistency with prior experiments. In our prior work, we had used doxycycline inducible MR1AGFP to define the optimal timing for expression of MR1AGFP^14^. We chose to induce MR1AGFP expression with doxycycline for 18 h either prior to transfection of MR1BRFP, or prior to flow cytometry or ELISpot, as this would ensure maximal expression of MR1AGFP, based on prior work^14^. To ensure that MR1AGFP expression was not artificially increased due to prolonged exposure to doxycycline, we showed that roughly 85% of these cells expressed MR1AGFP both before and after transfection with empty vector (Fig. 5B, right). Interestingly, transfection of pCI:MR1BRFP into Beas2B:MR1KO_Tet_MR1AGFP cells prior to the induction of MR1AGFP expression resulted in a 20% increase in MR1AGFP-negative cells, as compared to cells with doxycycline-induced MR1A expression prior to the transfection of pCI:MR1BRFP (Fig. 5C). This suggested that the expression of MR1B prior to the induction of MR1A could modulate total MR1A protein abundance. In contrast, the expression of pCI: MR1CRFP either pre or post induction of MR1A had no effect on MR1A protein production (Fig. 5D).

To assess the impact of MR1BRFP expression prior to induction of MR1AGFP expression on the recognition of microbial ligands, we repeated the experiment as described in Fig. 5B, and infected the cells with *M. smegmatis* (Fig. 5D). We observed that, compared to the no-doxycycline control, Beas2B_MR1KO:Tet_MR1AGFP transfected with an empty vector were capable of stimulating MAIT cell activation upon the administration of doxycycline (Fig. 5D). Upon transfection of MR1B in MR1A expressing cells, we observed that when MR1A and MR1B were both expressed, there was a 30% decrease in the activation of MAIT cells, as seen in prior experiments with MR1A expressed using the CMV promoter. However, we observed an approximately 50% inhibition of MAIT activation when MR1B was expressed prior to induction of MR1A protein, which might reflect the lower MR1A protein abundance under these conditions (Fig. 5D, left). Expression of pCI:MR1CRFP pre or post induction of MR1A expression did not impact MR1A-mediated expression or antigen presentation, indicating this was not a general consequence of transfection (Fig. 5E, 5F). Thus, prior expression of MR1B may inhibit the abundance of MR1A protein, either through competing for chaperones necessary for MR1A folding, or by competing for factors required for protein synthesis of MR1.

### Expression of MR1 splice variants in CD4/CD8 MR1-expressing thymocytes

**Figure 6:**
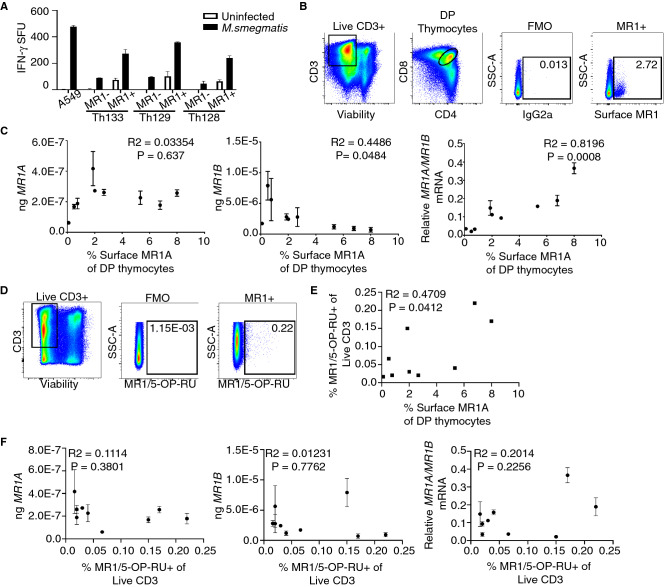
Double positive thymocyte expression of surface MR1A is associated with transcript expression and MAIT frequency in the thymus. MR1 + thymocytes were isolated using magnetic sorting based on MR1 expression (**A**) and used as antigen presenting cells following infection with *M.smegmatis* for 1 h at an MOI of 3 to stimulate MAIT production of IFN-γ, Error bars represent mean and standard deviation of duplicate wells from one experiment. T cell production of IFN-γ is represented as spot forming units (SFU). (**B**) Gating strategy for sorting MR1 + CD4 + CD8 + (DP) thymocytes from human thymus. Singlet cells were gated on live CD3 + cells. Live CD3 + cells (left) were gated on CD4 and CD8 DP positives (middle). Gate for MR1 + cells was set based on an IgG2a isotype control for MR1(third). An example plot of MR1 + cells based on this gating strategy is shown (right) (**C**) Absolute (left, middle), and relative (right) amounts of MR1A and MR1B transcript in DP MR1 + thymocytes versus surface expression of MR1 in 9 thymus donors. Pearson’s correlation was calculated and R^2^ and statistical significance is reported. (**D**) Gating strategy to identify MAIT cells in human thymus. Gates were set based on a negative control (FMO). Briefly, cells were stained with markers for viability, CD3, and the MR1/5-OPRU tetramer or the MR1/6-FP control tetramer (not shown). Singlet cells were gated on live CD3 + (left) and gates for MR1/5-OPRU tetramer + were set based on a negative control (FMO-middle). An example donor is shown on the right (Th97) (**E**). Frequency of MR1/5-OPRU tetramer + MAIT cells (from **D**) plotted versus surface MR1A expression in DP thymocytes (from **B**) from 9 donors. Pearson’s correlation was calculated and R^2^ and statistical significance is reported. (**F**) Same as (**C**), Expression of MR1A and MR1B transcript in DP thymocytes (**B**,**C**) is plotted versus frequency of MR1/5-OPRU tetramer staining MAIT cells (**D**). Pearson’s correlation was calculated and R^2^ and statistical significance is reported. Error bars for all represent mean and standard deviation of triplicate wells from one experiment.

Amongst PBMC, surface expression of MR1 has been difficult to observe in the absence of either exogenous or bacterially-derived antigen(s)^31^. We have previously shown that CD4 + CD8 + double positive (DP) thymocytes constitutively express high levels of cell-surface MR1^32^. It has been previously reported that the percentage of MR1-expressing thymocytes varies from 0.5 to 15% of DP thymocytes^32^. The role of MR1 expressing DP thymocytes in the selection of MAIT thymocytes is not well understood^33^. To determine whether MR1 + DP thymocytes are capable of presenting mycobacterial antigen to activate MAIT cells, we enriched for MR1-expressing DP thymocytes from human thymus using positive selection following surface staining for MR1A (Table 1). To determine if these cells could stimulate MAITs, the MR1 + enriched fraction as well as the MR1 depleted fraction were incubated for 1 h with *M. smegmatis* (MOI = 3) and tested for their ability to activate MAITs. As shown in Fig. 6A, the MR1-expressing DP thymocytes could activate MAIT cell production of IFNγ as compared to thymocytes not expressing cell surface MR1.

**Table 1:** Frequencies of MR1 + and MAIT populations in the thymus.

Donor	Prevalence of MR1 expressing thymocytes (% of DP thymocytes)	MR1 protein expression (gMFI MR1 + cells)	Prevalence of MAITs (% of 5-OP-RU Tetramer + of Live CD3)
90	5.33	1529	0.048
97	6.77	1,466	0.200
99	8.00	1,337	0.150
106	2.67	788	0.020
107	2.00	816	0.020
110	0.50	1,060	0.058
118	0.75	873	0.018
121	0.10	796	0.130
152	1.87	644	0.130

To assess whether relative expression of the MR1 isoforms is associated with prevalence of MR1A expressing thymocytes, we sorted MR1 expressing DP thymocytes from nine donors (Fig. 6B). We then measured expression of *MR1A* and *MR1B* mRNA by qRT-PCR, which we then compared to surface MR1A expression in these donors (Fig. 6C). We observe that there was not a strong association between *MR1A* mRNA and surface expression of MR1 (R^2^ = 0.03354, p = 0.637), but there was a significant association between *MR1B* transcript and the frequency of MR1 + DP thymocytes (R^2^ = 0.4486, p = 0.0484). Intriguingly, we observed a strong positive association (R^2^ = 0.8196, p = 0.0008) between relative *MR1A/MR1B* transcript and the frequency of MR1 + DP thymocytes.

To explore the relationship between DP MR1 expressing thymocytes and MAIT abundance in the thymus, we used the tetramer of MR1 bound to 5-OP-RU (MR1/5-OP-RU) to identify MAITs (Fig. 6D)^34^. Among CD3 thymocytes, we observed frequencies of MAIT cells, with variation across donors (0.01–0.45% of live CD3 + cells, Table 1). Intriguingly, the frequency of MR1 expressing DP thymocytes was associated with the frequency of MR1/5-OPRU + thymocytes (Fig. 6E) (R^2^ = 0.4709, p = 0.0412). However, the absolute amounts of *MR1A* and *MR1B* mRNA did not appear to be significantly correlated with frequency of MR1/5-OP-RU + thymocytes (Fig. 6F). These results suggest a potential role for alternative splicing of MR1 in contributing to selection and development of MAIT cells in the thymus.

## Discussion

In this report, we show that MR1B, an alternatively spliced form of MR1, inhibits mycobacterial antigen presentation to MAIT cells by the full-length variant, MR1A. Both MR1A and MR1B are ubiquitously expressed, but with considerable variation between both discrete cell and tissue types as well as individual donors. These results suggest that MR1B can serve to modulate the recognition of MR1 ligands by MR1A. We postulate that in addition to microbial recognition, MR1B could play a role in the prevention of unwanted MAIT cell activation, and in the development of MAIT cells.

These results raise a number of questions about the mechanism(s) by which MR1B mediates this inhibitory function. As we found that MR1B did not traffic to the cell surface following exposure to exogenous ligand, we believe that MR1B is not directly blocking TCR engagement with MR1A. Additionally, as MR1A and MR1B reside in the same intracellular compartments, we postulate that the inhibitory effect of MR1B could depend upon its proximity to MR1A. For example, MR1B may be competing for ligand, limiting the ability of MR1A to bind and present antigen to MAIT cells, or reducing MR1A folding and stability. Alternatively, MR1B could compete for binding to a molecular chaperone which is necessary for the loading and/or stabilization of the MR1A molecule following exposure to bacterial ligands, or for subsequent translocation of the loaded MR1A-ligand complex to the cell surface.

While coexpression of MR1A and MR1B resulted in decreased MAIT activation, we did not observe a decrease in MR1A protein abundance or surface expression. However, the co-transfection approach cannot replicate the variations in MR1A and MR1B expression and relative expression that we observed in our analysis of gene expression across cells and tissues. To address this, we have developed an MR1A expression system dependent on doxycyline, that in turn allows to vary the timing and abundance of MR1A relative to MR1B. Here, we found that expression of MR1B prior to MR1A resulted in a decrease in total MR1A protein, further inhibiting the presentation of ligands to MAIT cells. Further studies involving analysis of MR1B protein structure, ligand binding, or possibly its associated chaperones could would be logical next steps to elucidate the mechanism by which MR1B is acting to inhibit MR1A.

We found that both the abundance and relative expression of MR1A and MR1B varies considerably across tissues and donors. At present, the significance of this variation is not clear with regard to MAIT cell prevalence in tissues, their state of activation, and their ability to respond to MR1 antigens. Preliminarily, we note that tissues such as blood and spleen expressed relatively higher levels of *MR1A/MR1B*, while uterus, breast, and colon expressed lower *MR1A/MR1B* transcript. As these data were derived from mRNA from the whole tissue rather than specific cell types, the full significance of these data will require further investigation. Importantly, we observe that in unfractionated single cells prepared from the small intestine, we observed relative expression of *MR1A/MR1B* transcript that was similar to what we observed in our analysis of large datasets. This would suggest that different cell types within a given tissue can express markedly different levels of MR1 isoforms. However, the wide variation would lead us to speculate that the relative abundance of MR1B could be associated with diminished activation of tissue resident MAITs. This could have significance with regard for the ability to recognize microbial infection or be associated with a propensity for autoimmunity.

Alternative splicing is often regulated by genetic modifications such as changes in DNA methylation or single nucleotide polymorphisms (SNPs)^35,36^. Recently, an intronic SNP in *MR1* was shown to be associated with susceptibility to tuberculosis^37^. These results raise the possibility that regulation of MR1A and MR1B presentation is controlled at the level of gene expression. Alternately, we postulate microbial infection could alter expression of MR1 splice variants.

Alternative splicing is common and represents an important means of controlling gene expression. For instance, dysregulated splicing has been associated with both autoimmunity and inflammation^38–40^. However, the role of alternative splicing in the regulation of MHC and subsequent acquisition of adaptive immunity is incompletely explored. An α3 domain deletion mutant of HLA-A11 can promote evasion of NK cell mediated cytotoxicity of the HIV-infected cell^41^. Alternative splicing of the non-classical molecule HLA-G has been reported and is hypothesized to be associated with protection of the developing fetus^42,43^. These reports suggest that a putative role for alternative splicing is to control immune cell activation and therefore target immune responses to microbial infection. A recent study suggested that viral infection downregulates surface expression of MR1 and thereby presentation of MR1-bound antigen to MAIT cells, through the HSV-1 protein US3^44^. The mechanism of this is unknown but suggests that impeding MR1 function can further regulate MAIT cell activity. Here, the relationship of MR1 splice variants in the context of both infection and autoimmunity remains to be explored.

Our results demonstrate an association between the relative *MR1A/MR1B* expression and prevalence of MR1A-expressing CD4 + CD8 + thymocytes. CD1d expressing DP thymocytes have been shown to contribute to the selection of iNKT cells, and MR1 + DP thymocytes have been suggested to play a similar role in the selection of MAIT cells in the thymus^45,46^. Furthermore, we find that the prevalence of MAIT cells in the thymus was associated with MR1A-expressing DP thymocytes. While the frequency of MR1A expressing DP thymocytes varied, cell surface of MR1 protein was relatively constant between donors. Therefore, it is clear that the cell surface expression of MR1 is not strictly dependent on the relative abundance of the MR1A and MR1B splice variants. Recent work by Legoux and colleagues demonstrated that intrathymic development of murine MAIT cells could be governed, in part, by metabolites produced by commensal bacteria presented by MR1^47^. Therefore, mechanisms regulating MR1 expression and capacity to bind and present antigen, could potentially also contribute to thymic development of MAIT cells. We observed that relative *MR1A/MR1B* expression on DP thymocytes was associated with the prevalence of MR1 + DP thymocytes. Additionally, the prevalence of MR1 + DP thymocytes was associated with that of MAIT precursors in the thymus. How DP MR1 expressing thymocytes function in MAIT development and the role of alternative splicing in this development will require detailed in vitro modeling. One limitation of our study is that we did not have matched PBMC from our thymus donors, and therefore, we were not able to establish the relationship of intrathymic MR1 expressing cells and the prevalence of MAITs in the blood. Taken together, our results suggest a potential role of alternative splicing in modulating presentation of microbial ligand to MAIT cells.

## Materials and methods

### Statement

All experiments and methods were performed in accordance with relevant guidelines and regulations.

### Reagents and antibodies

Doxycycline (Sigma-Aldrich. St. Louis, Missouri) was resuspended to 2 mg/mL in sterile water and was used at 2 µg/mL. 6-formylpterin (Schirck’s Laboratories, Bauma, Switzerland) was resuspended to 3 mg/mL in 0.01 M NaOH. Control vehicle used was 0.01 M NaOH**.** Antibodies utilized are listed in Table 2

**Table 2:** Antibodies used in flow cytometry surface staining.

Antibody Name	Fluorophore	Manufacturer	Clone
CD4	PE Cy7	BioLegend	RPA-T4
CD326	APC	BioLegend	9C4
CD14	FITC	Biolegend	HCD14
CD20	FITC	BioLegend	2H7
CD45	FITC	Biolegend	30-F11
Live-Dead	Aqua	ThermoFisher	
MR1/5-OP-RU tetramer	PE	NIH Tetramer Core (Emory)	
MR1/6FP tetramer	PE	NIH Tetramer Core (Emory)	

### Microorganisms and preparation of APCs

*M. smegmatis* and *M. bovis* Bacillus Calmette-Guérin (BCG) were utilized from frozen glycerol stock. For short-term *M.smegmatis* infection, cells were infected for 1 h in a 96 well ELISPOT MSHA nitrocellulose plate at the MOI indicated in the figure. For overnight *M.smegmatis* infection, cells were infected for 16 h at an MOI of 3. For thymocyte infection with *M. smegmatis*, thymocytes were infected at an MOI of 3 for 1 h. For BCG infection, adherent cells were infected overnight at a MOI of 15. BCG infected cells were harvested and added to the ELISPOT assay at the amount indicated in the figure. To generate *M. smegmatis* supernatant, *M. smegmatis* was cultured with shaking for 24 h then pelleted. The supernatant was passed through a 0.22 µm filter to remove any bacteria. The supernatant was utilized at the indicated volume in an ELISPOT assay.

### Expansion of T-cell clones

T-cell clones were cultured in the presence of X-rayed (3,000 cGray using X-RAD320, Precision X-Ray Inc.) allogeneic PBMCs, X-rayed allogeneic LCL (6,000 cGray) and anti-CD3 monoclonal antibody (20 ng ml^−1^; Orthoclone OKT3, eBioscience, San Diego, USA) in RPMI 1,640 media with 10% human serum in a T-25 upright flask in a total volume of 30 ml. The cultures were supplemented with IL-2 on days 1, 4, 7 and 10 of culture. The cell cultures were washed on day 5 to remove soluble anti-CD3 monoclonal antibodies.

### IFN-γ ELISPOT

A MSHA S4510 96 well nitrocellulose-backed plate (Millipore, bought via Fisher Scientific, Waltham, USA) was coated overnight at 4 °C with 10 μg ml^−1^ solution of anti-IFN-γ monoclonal antibody (Mabtech clone 1-D1K, Mabtech, Stockholm, Sweden) in a buffer solution of 0.1 M Na_2_CO_3_, 0.1 M NaHCO_3_, pH = 9.6). Then, the plate was washed three times with sterile PBS and blocked for 1 h at room temperature with RPMI 1,640 media containing 10% heat-inactivated HS pool. Then, the APCs and T cells were prepared as described above and co-incubated overnight. Antigen presenting cells and antigen used are noted in the relevant figure. T-cell clones were added at 5 × 10^3^ per well. The plate was incubated overnight at 37 °C and then washed six times with PBS containing 0.05% Tween. The plate was then developed as previously described and analyzed using an AID ELISPOT reader (Strassberg, Germany)^5^.

### Human subjects

All samples were collected, and all experiments were conducted under protocols approved by the institutional review board at Oregon Health and Science University. PBMCs were obtained by apheresis from healthy adult donors with informed consent. De-identified lungs, upper airway, or small intestine were obtained from the Pacific Northwest Transplant Bank (PNTB, Portland, OR) (Table 3) with informed consent from next of kin and/or parent or guardian if < 18. Deidentified thymuses were obtained as medical waste from an exempt IRB protocol from children undergoing cardiac surgery at Oregon Health and Science University Doernbecher Children’s Hospital. The majority of children were no less than 4 mo. and no more than 4 years old. Informed consent was obtained from parent or guardian for all donors < 18.

**Table 3:** Table of airway, lung, and small intestine donors.

Donor	Gender	Cause of death	Mechanism of injury	Age	Smoking history?
OD24	Male	Head trauma	Blunt injury	22 months	No
OD48	Male	cerebrovascular/stroke	ICH/stroke	44	Yes
OD63	Male	cerebrovascular/stroke	ICH/stroke	57	No
OD78	Female	Cerebrovascular/stroke	ICH/stroke	69	No
OD83	Male	Cerebrovascular/stroke	ICH/stroke	65	No
OD88	Female	Anoxia	Asphyxiation	29	No
OD92	Female	Cerebrovascular/stroke	ICH/stroke	48	Yes
OD93	Female	Head trauma	Blunt injury	52	Not indicated
OD97	Male	Head trauma	Gunshot wound	55	Not Indicated
OD101	Female	Cerebrovascular/stroke	ICH/stroke	64	No
OD111	Female	Anoxia	Not indicated	22	No
OD114	Male	Cerebrovascular/stroke	ICH/stroke	56	Not indicated
OD119	Female	Cerebrovascular/stroke	ICH/stroke	58	No
OD120	Female	Anoxia	Drug intoxication	39	No
OD121	Female	Anoxia	Not indicated	6	No
OD123	Male	Cerebrovascular/stroke	ICH/stroke	39	No

*Determination of smoking history made based on > 20 pack/year history.

### Cell lines

BEAS-2B and A549 cell lines was obtained from ATCC. A549:MR1_KO were generated as described previously^29^. All cell lines were cultured in DMEM (Gibco via ThermoFisher Scientific, Waltham, MA) supplemented with L-glutamine and 10% heat inactivated FBS. All cell lines were confirmed to be mycoplasma free.

### Generation of lentivirus particles

Lentivirus production was performed as described previously^48^. Briefly, lentiviruses were produced by co-transfection of HEK 293T cells with the lentiviral vectors that contained the gene of interest (pCDH-sgRNA or pCW-Cas9) and the packaging plasmids (psPAX2 and pMD2.G). Transfection was performed with Lipofectamine 3,000 (Thermo Fisher, Waltham, USA) according to manufacturer’s instructions. Cells were cultured in DMEM supplemented with 10% FBS. 48 h after transfection, lentivirus-containing supernatants were harvested, centrifuged for 5 min at 1,250 rpm and filtered through a 0.45 µm filter. The Beas2B_MR1KO cell line was transduced with a lentivirus expressing pDMLV_Tet_MR1AGFP by spinoculation. Transduced cells were exposed to doxycycline overnight to promote expression of MR1AGFP and subsequently sorted on an InFlux cell sorter based on GFP expression. Cells were subsequently cultured, validated for MR1AGFP expression by flow cytometry, and utilized for further experiments.

### Generation of a Beas2B MR1 knockout cell line

CRISPR/Cas9-mediated genome-editing for MR1 was performed as described previously^48^. Wildtype Beas2B cells were seeded in a 6 well plate and transduced by spinoculation with a lentivirus encoding the CRISPR/Cas9 gene with guide RNA specifically targeted to MR1 that had been previously used to generate an A549 MR1 knockout cell line^29^. Following this, limiting dilution was performed on transduced cells. Cells were grown in DMEM media containing 10% fetal bovine serum and 1% gentamicin antibiotic. Three clones were validated as knocked out for MR1 protein expression, as well as functionally unable to stimulate MAIT clones, and cryopreserved in 90% fetal bovine serum/10% DMSO. One clone was selected and utilized for all future experiments (Beas2B:MR1_KO).

### Human tissue sources of antigen presenting cells

PBMCs were isolated from the peripheral blood of healthy donors using Ficoll-Paque gradients.

Monocyte-derived DCs and macrophages were generated by the method of Romani^28^. PBMCs obtained by apheresis were resuspended in 2% human serum in RPMI and were allowed to adhere to a T-75 flask at 37 °C for 1 h. After gentle washing twice with PBS, nonadherent cells were removed and 10% human serum in RPMI containing 30 ng ml^−1^ of IL-4 (Immunex, Seattle, USA) and 30 ng ml^−1^ of granulocyte–macrophage colony-stimulating factor (Immunex, Seattle, USA) was added to the adherent cells to generate dendritic cells. For generation of macrophages cells, adherent cells were incubated in 10% human serum in RPMI for 1 h. The cells were X-rayed with 3,000 cGray using X-RAD320 (Precision X-Ray Inc, North Branford, USA) to prevent cell division. After 5 days, cells were harvested with cell-dissociation medium (Sigma-Aldrich, Gillingham, UK) and used as APCs in assays.

Lung and small intestine single cell suspensions were prepared from recently deceased donor tissue. Characteristics of deceased donors are listed in Table 3 Lung parenchyma and duodenal mucosa were digested for 30 min at 37ºC in DMEM buffer (Gibco via ThermoFisher, Waltham, US) supplemented with PSF antibiotics (Sigma, St, Louis, USA), elastase (15 µg/mL, Worthington), trypsin I (1.5 µg/mL, Sigma, St. Louis, USA), DNase I (45 µg/mL, Roche, Basel, Switzerland). The suspension was dissociated using a GentleMACS dissociator (Miltenyi, Bergisch Gladbach, Germany), passed through metal mesh sieve filters (size 40 then 60, Sigma), and through nylon cell strainers (100 μm then 40 µm, BD Falcon, Franklin Lakes, USA). The resulting cell suspension was washed in RPMI supplemented with 10% heat inactivated human serum and cryo-preserved in heat-inactivated fetal bovine serum with 10% DMSO.

Large airway epithelial cells were generated as described previously from the upper airway of human donors and cryopreserved in heat-inactivated fetal bovine serum with 10% DMSO^2^.

Thymocytes: Thymus tissue was ground in a GentleMACS dissociator with of DMEM plus 10% FBS to form a single cell suspension. The suspension was cryopreserved at 2–3 × 10^8^ cells/ml in a 90% FBS/10% DMSO freezing solution with a post-thaw viability of approximately 50%

### RNA isolation, cDNA synthesis and qPCR analysis

Total RNA was isolated using TRIZOL (Life Technologies, Carlsbad, USA) and phenol chloroform extraction followed by an RNAeasy Kit (Qiagen, Venlo, Netherlands). cDNA was synthesized using a High Capacity cDNA Reverse Transcription Kit (ThermoFisher Scientific, Waltham, USA). qPCR was performed using SYBRGreen Power Master Mix (ThermoFisher Scientific, Waltham, USA) on a Step One Plus Real-Time PCR System (Applied Biosystems, Foster City, USA). Primers were designed to be specific for each gene assay and generated by the DNA services core and IDT Technologies. The absolute quantification method was used using by generating a standard curve with a plasmid specific for each gene assayed. Primer sequences are noted in Table 4.

**Table 4:** Primer pairs used for qRT-PCR.

Primer name	Sequence
MR1AFwd	AGGGGTTACAGCTCTCTTCTG
MR1ARev	TTGATGCCCACGCCTG
MR1BFwd	GCTCACACCATCAAGCAG
MR1BRev	GAGACAGCTTTCATCACAAGAGG
MR1CFwd	GCTCCATTTTGCTCTGTTCTTT
MR1CRev	TCCTGATCTTCAATAAAGACA
GAPDHFwd	GTCTCCTCTGACTTCAA
GAPDHRev	ACCACCCTGTTGCTGTA

### Construction of MR1B_RFP and MR1C_RFP

We utilized our previously described pCI construct for all transient transfections^12^. A gene encoding MR1B_RFP or MR1C_RFP was ligated into this construct using EcoRI and KpnI, to create pCI_MR1B_RFP or pCI_MR1C_RFP. Confirmatory sequencing was performed. Restriction enzymes and ligation kit were obtained from New England Biolabs (Ipswich, USA). PCR and gel purification kits were obtained from Qiagen (Venlo, Netherlands).

### Plasmid transfections

Transfection of Beas2B or A549 cells using plasmids was done using an Amaxa Nucleofector, Kit T solution (Lonza, Basel, Switzerland), and programs G-016 according to manufacturer’s protocol. For co transfections, a 2:1 ratio of MR1B:MR1A was used unless otherwise specified, and the amount of MR1A was kept fixed at 2.5 ug. For single transfections, 2.5 ug of plasmid was transfected. A pCI empty vector was used as a control for all transfections. Confirmation of transfection was performed using flow cytometry on a BD Fortessa to detect either GFP (pCI_MR1AGFP) or RFP (pCI_MR1BRFP or pCI_MR1CRFP). All analyses were performed using FlowJo software (TreeStar, Ashland, OR, USA).

### MR1 surface stabilization

BEAS-2B:MR1_KO cells, either untransfected, or transfected with pCI empty vector, pCI_MR1AGFP, and/or pCI_MR1BRFP, were plated into 6 well tissue culture plates overnight. The next day, the cells were treated with 100 µM 6-FP versus an equivalent volume of 0.01 M NaOH. After 16 h, the cells were harvested on ice and split into two groups for primary staining and isotype control staining. Primary staining was done with an antibody against MR1 (Clone 26.5, gift from Ted Hansen, biotinylated by Biolegend, San Diego, USA) at 1:100 for 40 min on ice in the presence of 2% human serum, 2% goat serum, and 0.5% FBS. Biotinylated mouse anti IgG2A (Biolegend, San Diego, USA) served as the isotype control. After washing, streptavidin-Alexa 647 (ThermoFisher Scientific, Waltham, USA) was added for 40 min on ice. Cells were washed, fixed in 1% PFA and analyzed by flow cytometry on a BD Fortessa, or BD Symphony and data were analyzed on FlowJo (TreeStar, Ashland, USA).

### Fluorescence microscopy

BEAS-2B:MR1_KO cells were transfected with MR1A_GFP or MR1B_RFP and plated into 1.5 mm glass bottom chamber slides (Nunc, Roskilde, Denmark; ThermoFisher Scientific, Waltham, USA) and incubated at 37° C and 5% CO_2_. After 48 h, live images were acquired on a high-resolution wide field CoreDV system (Applied Precision, Pittsburgh, Pennsylvania) with a Nikon Coolsnap ES2 HQ. For 6FP surface stabilization assay, cells were transfected as above, and administered 50 uM 6FP or an equivalent volume of 0.01 M NaOH for 16 h. Cells were fixed for 20 min in 4% PFA and stained with DAPI (ThermoFisher Scientific, Waltham, USA) to visualize nucleus before acquisition on a wide field CoreDV system. Each image was acquired as Z-stacks in a 1,024 × 1,024 format with a 60 × objective (NA 1.42).

### Dynamics of MR1A and MR1B expression

pCI_MR1BRFP or pCI_MR1CRFP was transfected into Beas2B_MR1KO cells overexpressing a Tet_MR1AGFP lentivirus either 24 h prior to or 16 h post induction of MR1A expression. Flow cytometry was performed as described earlier to measure MR1AGFP expression or MR1BRFP expression using a BD Symphony and data were analyzed using FlowJo software. For functional analyses, cells were harvested on ice, infected 1 h with a titrating dose of M. smegmatis, and utilized as antigen presenting cells (5 × 10^3^/well) in an ELISPOT assay following incubation with an MAIT clone (5 × 10^3^/well). The ELISPOT was set up and developed as described above.

### Flow cytometry staining and FACS sorting

Lung and small intestine cell suspensions generated as described earlier were thawed, washed with PBS twice and blocked in FACS buffer. 2e06 cells were stained with antibodies against surface CD45, CD326 (EPCam), CD14, and CD20, for 30 min on ice in the dark, as well as Live/Dead Fix Aqua for viability. Cells were harvested on ice, washed in PBS, resuspended in FACS buffer, and sorted on a BD InFlux sorter for Live, CD45- CD14-, and CD20- CD326 + cells in order to isolate CD326 + epithelial cells. Cells were sorted into TRIZOL for immediate RNA isolation and subsequent quantitative real time PCR analysis. Sorting of MR1 + DP thymocytes: Thymocytes were thawed and blocked with FACS buffer, and 2e06 cells were stained with antibodies for the surface markers CD3, CD4, CD8, and MR1-biotin (clone 26.5), or the isotype control (IgG2a-biotin) for 30 min on ice in the dark. Cells were washed twice with cold PBS and stained with streptavidin-PE (1:2000 dilution) for 15 min in the dark. Cells were washed twice and resuspended in FACS buffer and sorted on a BD InFlux Sorter for Live, CD3 + CD4 + CD8 + MR1 expressing thymocytes, which was confirmed based on an isotype control for MR1. Cells were sorted into TRIZOL for immediate RNA isolation and subsequent quantitative real time PCR analysis.

For quantifying the frequency of MAIT thymocytes, thymocytes were thawed, blocked with FACS buffer, and 2e06 cells were incubated with the MR1/5-OP-RU tetramer or the MR1/6FP tetramer (1:500 dilution, NIH Tetramer Core facility, Emory, USA), for 45 min at RT in the dark. Following this, antibodies to the surface markers CD3, CD4, and CD8 and Live/Dead Fix Aqua viability stain were added at manufacturer recommended concentrations. Cells were incubated at RT in the dark for 15 min, washed twice with PBS, and fixed with 1% paraformaldehyde. Acquisition was performed on a BD Symphony flow cytometer and data were analyzed using FlowJo software (TreeStar, Ashland, USA). All antibodies utilized are listed in Table 4.

### GTEx analysis

Figure 1 depicts the approximate ratio of exon 4 inclusions to skips in *MR1* across normal tissue RNA-seq samples comprising the GTEx project^26^. To generate this figure, we first used Snaptron to query GTEx RNA-seq samples for numbers of reads spanning each of three exon-exon junctions, whose *hg38 *coordinates are (a) chr1:181,050,287–181,052,234, (b) chr1:181,052,511–181,053,572, and (c) chr1:181,050,287–181,053,572. Junctions (a) and (b) point to inclusion of exon 4, while junction (c) points to exclusion of exon 4. For a given sample, we averaged the numbers of reads spanning junctions (a) and (b) and divided the result by the number of reads spanning junction (c) to estimate the ratio of exon 4 inclusion to exclusion^25^. We subsequently used *Mathematica* 10.4 to plot results across samples. Scripts to reproduce Fig. 1 are available at https://github.com/nellore/mr1.

### Data analysis

Data were analyzed and plotted using Prism 7 GraphPad Software (La Jolla, California). Statistical significance was determined using unpaired Student’s two-tailed *t*-test, unless otherwise indicated. For comparison of DP MR1 + thymocyte gene expression with frequency of DP thymocytes or of MAIT thymocytes, Pearson’s correlation calculation was performed. For comparisons of functionality between transfected antigen presenting cells, linear regression analysis was performed and significant differences between slopes and y-intercepts were measured and reported. Error bars in the figures indicate the standard deviation, standard error of the mean, or the data set range as indicated in each figure legend. *P* values < 0.05 were considered significant (*P < 0.05; ** *P* < 0.01; *** *P* < 0.001).

## Supplementary information

Supplementary Information.

## Data Availability

The datasets analyzed during the current study are available from the publicly available GTex repository and can be accessed through the dbGap Study Accession number: phs000424.v8.p2. Scripts to reproduce Fig. 1 for the Snaptron analysis are are available at https://github.com/nellore/mr1.
