# Supplementary material for: Alternative splicing of MR1 regulates antigen presentation to MAIT cells
Source: Sci Rep. 2020 Sep 22;10:15429. doi: 10.1038/s41598-020-72394-9 (PMC7508857; doi:10.1038/s41598-020-72394-9)
Supplement: Supplementary file 1 — Supplementary Information. [file 41598_2020_72394_MOESM1_ESM.docx]

**Supplementary Information**

**Alternative splicing of MR1 regulates antigen presentation to MAIT cells**

Gitanjali A. Narayanan^1^, Abhinav Nellore^1,2^, Jessica Tran^3^, Aneta H. Worley^3^, Erin W. Meermeier^4^, Elham Karamooz^3,4^, Megan Huber^3^, Regina Kurapova^3^, Fikadu G. Tafesse^5^ , Melanie J. Harriff ^3,4,5^ David M. Lewinsohn*^3,4^

1. Department of Biomedical Engineering, Oregon Health and Science University, Portland, OR, USA
2. Department of Computational Biology, Oregon Health and Science University, Portland, OR, USA
3. VA Portland Health System, Portland, OR, USA
4. Department of Pulmonary and Critical Care Medicine, Oregon Health and Science University, Portland, OR, USA
5. Department of Molecular Microbiology and Immunology, Oregon Health and Science University, Portland, OR, USA

*Correspondence to David Lewinsohn: lewinsod@ohsu.edu

**
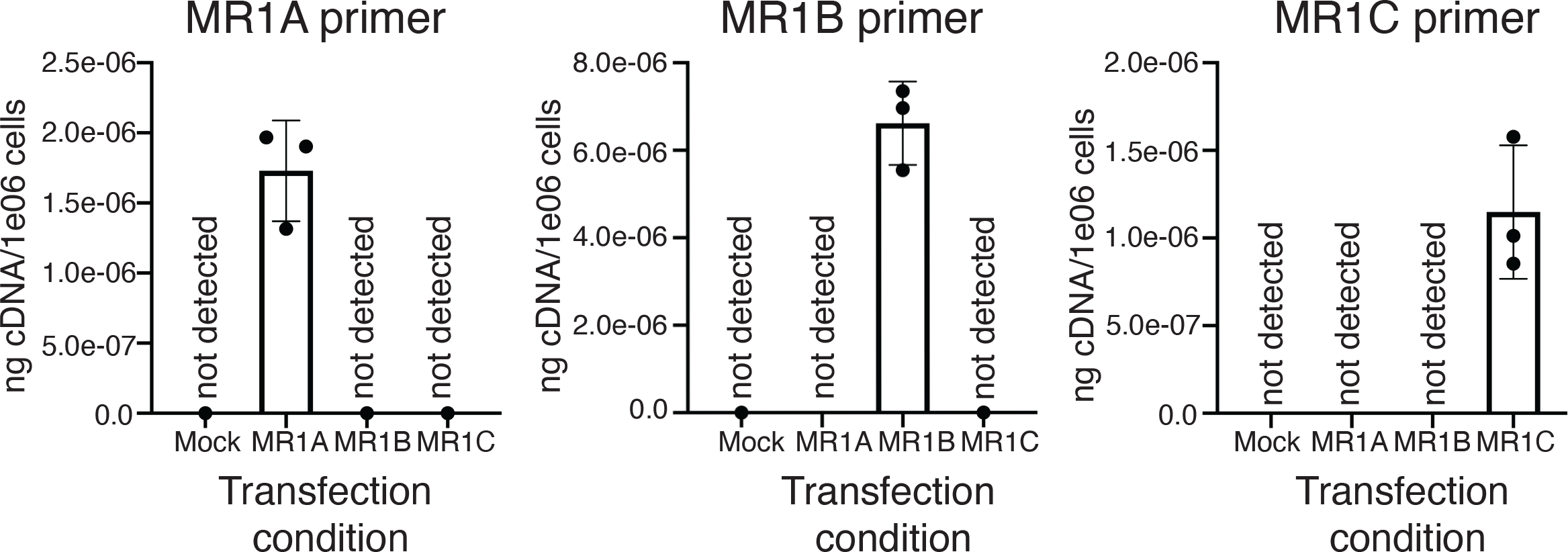
**

**Supplemental Figure 1: Validation of MR1 splice variant primers**

Beas2B_MR1:KO cells were transfected by Amaxa (Lonza) with plasmids encoding the pCi Empty Vector (Mock), MR1AGFP, MR1BRFP, or MR1CRFP, at manufacturer recommended quantities. 48h post transfection, cells were counted, and mRNA was isolated from 1e06 cells and converted to cDNA. qRT-PCR was performed on cells using the indicated primers (Primer sequences in Table 3), and normalized to a standard curve generated for each gene using serial dilutions of the indicated plasmid. Undetectable transcript is indicated. Experiment was performed in triplicate and data represent mean+SD of the mean


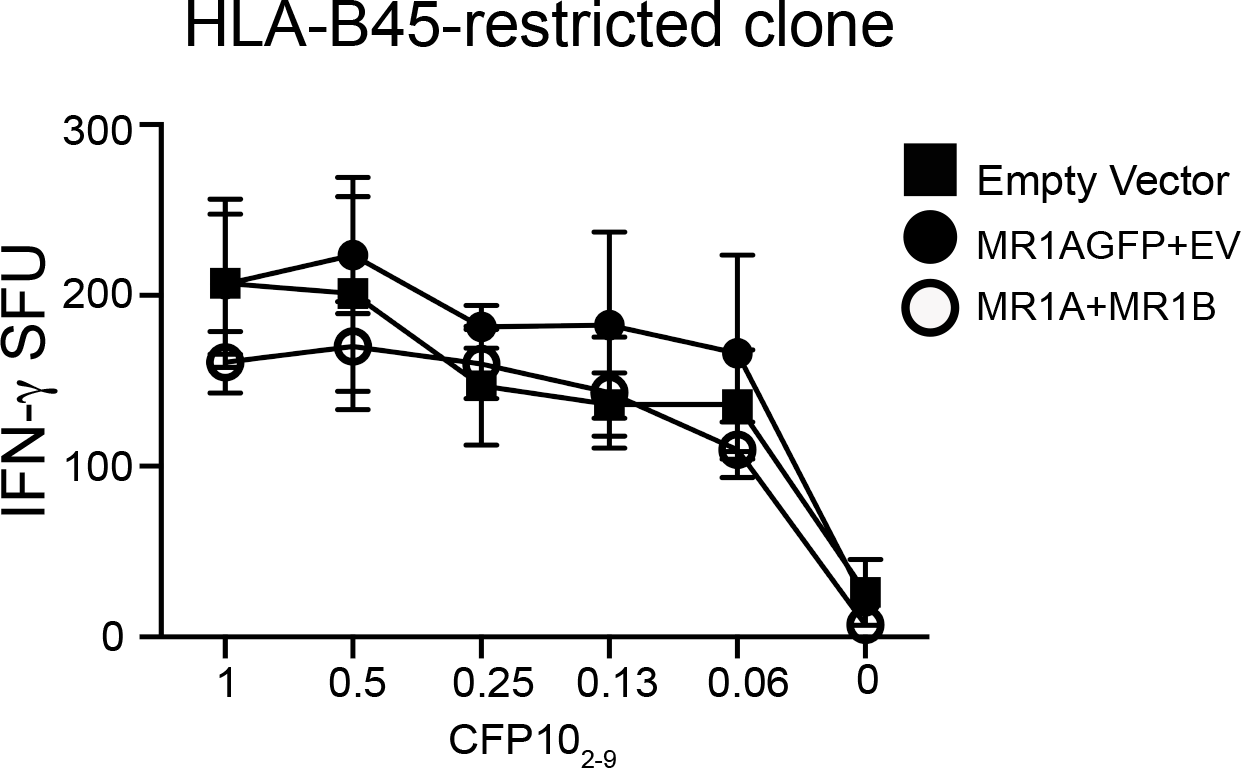


**Supplemental Figure 2: Cotransfection of MR1B with MR1A does not inhibit HLA-B45 dependent antigen presentation**

Beas2B_MR1:KO cells were transfected either with the pCI empty vector, or pCi:MR1AGFP + the pCI empty vector, or pCI:MR1AGFP + pCI:MR1BRFP for 48h. Cells were harvested and 1e03 cells were subsequently utilized as antigen presenting cells to present peptide at the indicated concentration to HLA-B45-restricted T cell clones. T cell production of IFN-g is measured on the y-axis. Experiment was performed twice in with triplicate wells per condition, and error bars represent mean+SD of 6 replicate wells.

**
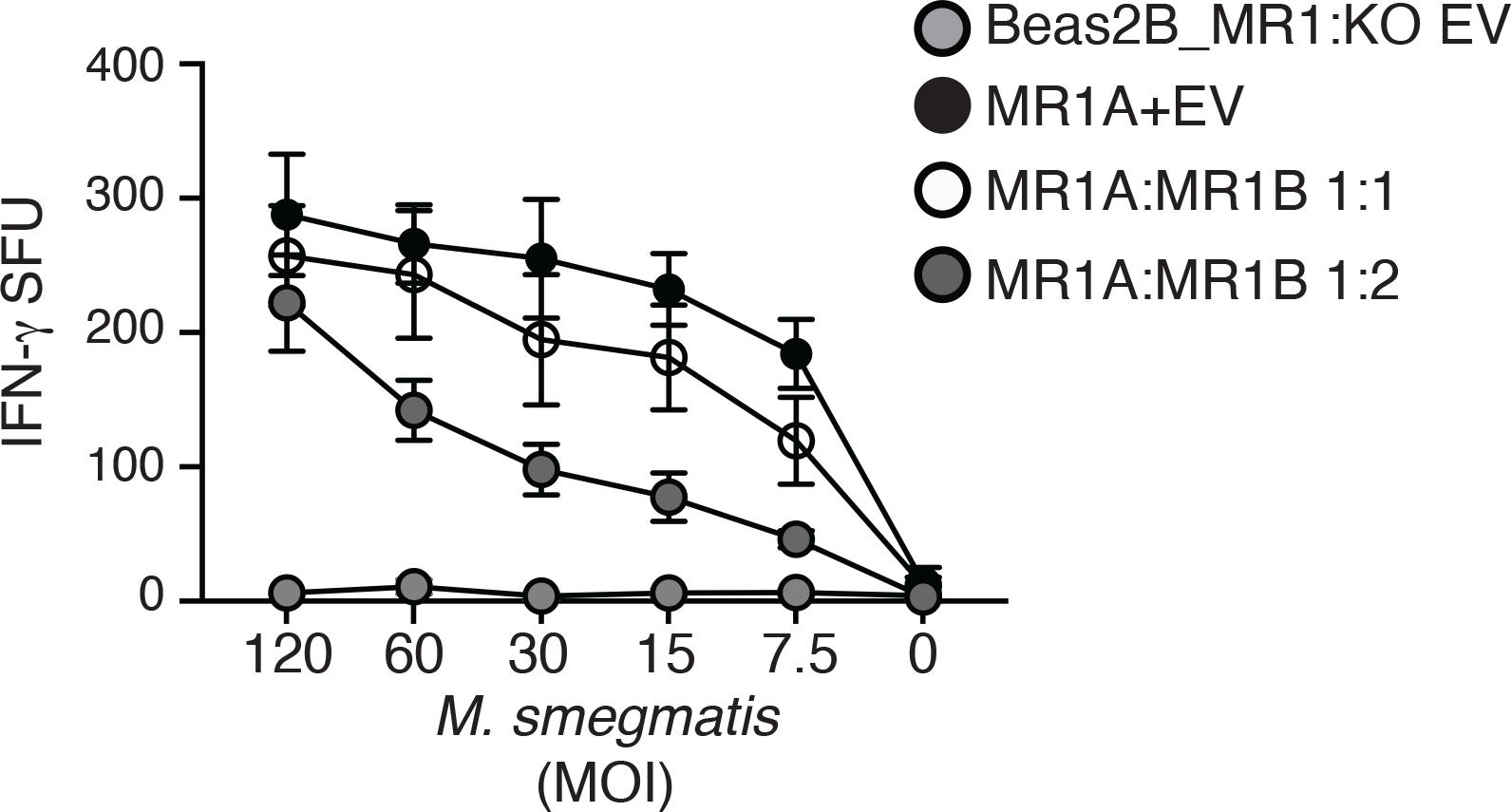
**

**Supplemental Figure 3: Validation of MR1B mediated antagonism**

Beas2B_MR1:KO cells were transfected with plasmids expressing pCI:MR1AGFP at a fixed amount (2.5 ug/transfection), and pCI:MR1BRFP at either 1:1 ratio or 2:1 ratio of MR1B to MR1A. A pCI empty vector was utilized as a control. Cells were incubated following transfection for 48h and subsequently harvested and utilized as antigen presenting cells (10000 APC/well) to activate MAIT cell clones following infection with *M. smegmatis* at the indicated MOI. MAIT cell production of IFN-g was assessed by ELISpot. Experiment was performed >3 times in duplicate with similar results, data represent mean and standard deviation of two independent experiments.
